# Analysis of Volatile Compounds and Flavor Fingerprint Using Gas Chromatography–Ion Mobility Spectrometry ( GC-IMS) on *Crassostrea gigas* with Different Ploidy and Gender

**DOI:** 10.3390/molecules28114475

**Published:** 2023-05-31

**Authors:** Jingjing Fu, Youmei Sun, Mingxian Cui, Enshuo Zhang, Luyao Dong, Yanchun Wang, Weijun Wang, Zan Li, Jianmin Yang

**Affiliations:** 1 School of Agriculture, Ludong University, Yantai 264025, China; fjj000117@163.com (J.F.); sunyoumei4554@163.com (Y.S.); cuimingxian666@163.com (M.C.); zenshuo1998@163.com (E.Z.); wycyc@163.com (Y.W.); lizanlxm@163.com (Z.L.); 2 College of Fisheries and Life Science, Shanghai Ocean University, Shanghai 201306, China; dly6568@163.com

**Keywords:** *Crassostrea gigas*, gas chromatography–ion mobility spectrometry (GC-IMS), ploidy, volatile, flavor

## Abstract

In this study, GC-IMS was used to analyze the volatile component and flavor profiles of *Crassostrea gigas* individuals of different ploidy and gender. Principal component analysis was used to explore overall differences in flavor profiles, and a total of 54 volatile compounds were identified. The total volatile flavor contents in the edible parts of tetraploid oysters were significantly higher than in diploid and triploid oysters. The concentrations of ethyl (E)-2-butenoate and 1-penten-3-ol were significantly higher in triploid oysters than in diploid and tetraploid oysters. In addition, the volatile compounds propanoic acid, ethyl propanoate, 1-butanol, butanal, and 2-ethyl furan were significantly higher in females than in males. The volatile compounds p-methyl anisole, 3-octanone, 3-octanone, and (E)-2-heptenal were present in higher levels in male than in female oysters. Overall, different ploidy and gender of oysters are connected with different sensory characteristics, providing new insights for understanding the flavor characteristics of oysters.

## 1. Introduction

Oysters belong to the Mollusca phylum, bivalve class, and pearl shell order. They are the most cultured shellfish globally, and are important marine resources. Oysters exhibit rich nutritional value [1] and are essential aquatic products that are incorporated into healthy diets [2]. Their meat is rich in proteins, fatty acids, and other compounds, leading to their widespread recognition of being delicious and their wide consumption by humans. The freshness and hygiene of oysters meet international standards and can thus be directly eaten raw [3]. Most oysters aquacultured in North China are Pacific oysters (*Crassostrea gigas*). The development of diploid oysters is seasonal, because propagation and ovulation occur in the summer, resulting in very thin gonads of diploid oysters after ovulation, which then affects their taste. For example, Qin et al. observed that the biochemical composition, nutritional value, and taste of triploid Hong Kong oysters (*C. hongkongensis*) were better than those of diploid oysters [4]. Compared with diploid oysters, triploid oysters are infertile, which reduces the energy loss caused by gonadal development [5], thereby leading to biological and economic advantages [6] that can improve meat quality [4]. Consequently, many aquaculture industries choose excellent quality triploid oysters, with the proliferation of triploid oysters becoming a top priority for the aquaculture industry. To prevent the release of a second polar body during fertilization, chemical [7] and physical methods are usually used to obtain triploid oysters [8]. However, chemicals used in chemical methods can harm experimenters due to improper operation, while triploids obtained using the physical method are not generated at a 100% induction rate [9]. However, stable and reliable triploid oysters can be produced by crossing a tetraploid male oyster gamete with a diploid female oyster gamete [8,10,11]. Moreover, tetraploid oysters can be successfully produced by self-breeding [12]. Concomitantly, some studies have observed that the biochemical compositions of oysters change, based on gonad development [13]. Li et al. showed that glycogen provides the primary energy for the occurrence of oyster gametes, and gradually decreases with gonadal development [14]. In addition, oocytes are stalled in the meiosis M1 stage before spawning [15,16], while sperm can complete two meiosis stages [17], inevitably leading to differences in energy expenditure and structural components between female and male oysters. It is consequently possible that the specific meiotic modes of oysters may result in large differences in volatile compound compositions between male and female oysters.

Volatile flavor compounds (VFCs) play important roles in the sensory and quality characteristics of oysters. Some studies have shown that vacuum hydrodistillation can characterize the key odor compounds of *C. gigas* in fresh oyster, which is more suitable for describing its sensory characteristics [18]. Pennarun et al. demonstrated that the sensory qualities and volatile compounds of oysters are altered by a microalgae diet [19]. In addition, the formation of volatile flavors in oysters is closely related to various chemical reactions involving lipids, proteins, and sugars [2]. For example, Ma et al. reported that hydrocarbons are the most prominent volatile flavor substances in oysters. Among these, 3-octanone appears to be the primary volatile compound in oysters, and produces a mushroom-like aroma [2]. Lin et al. investigated diploid and triploid oyster-free amino acid, inosine monophosphate, succinate, trimethylamine oxide, and betaine levels, revealing that combined chemical analysis with sensory evaluation is essential for accurately assessing the tastes of oysters [6]. Houcke et al. assessed differences in the volatile organic compounds (VOCs) produced by *Ostrea edulis* and *C. gigas* in two separate areas of the Netherlands, demonstrating that the primary volatile compound in *O. edulis* was 3-cyclohexene-1-ethanol, but 1, 5-octandiene-3-ol in *C. gigas* [20]. Sheng Liu et al. also speculated that aldehydes might be the characteristic flavor compounds in *C. sikamea* [21]. Van Houcke et al. found that different varieties, breeding areas and harvesting seasons all had important effects on the volatile components of the oyster. The difference between the January- and February-harvest volatile components was mainly caused by 1-pentene-3-ol, (E, Z)-2,6-nondienal and heptal [20]. Cochet.M. et al. showed that the growth cycle and breeding environment of oysters have a certain influence on its nutritional composition and quality, which in turn also affects its volatile flavor components [22]. There is no relevant literature to prove that there is odorant in oysters in a conjugated form which can be released because of the interaction with the tongue. Nevertheless, few studies have reported differences in VFCs between various oysters with different ploidy and sex characteristics. Of the few, Qin et al. investigated differences in biochemical components of three oyster types [13]. Nevertheless, few studies of volatile flavor components have been conducted by comparing male and female oysters. Consequently, the aim of this study was to explore differences in VFC profiles for oysters with different ploidy levels and sexes via GC-IMS and the development of fingerprint maps. These results provide new insights for understanding the flavors of different ploidy oysters.

## 2. Results and Discussion

### 2.1. GC-IMS Profiles of Male and Female Oysters with Different Ploidy Levels

**Figure 1:**
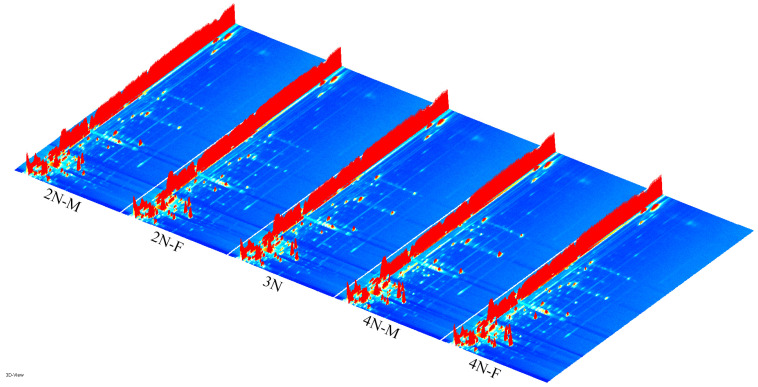
Three-dimensional spectrum of the volatile components for diploid male oyster (2N-M), diploid female oyster (2N-F), triploid oyster (3N), tetraploid male oyster (4N-M) and tetraploid female oyster (4N-F), evaluated using GC-IMS. Note: the bright spot denotes a volatile component, and its hue spans from blue to red, signifying the concentration of the compound from less to greater. The *x*-axis, *y*-axis, and *z*-axis indicate the migration time (normalized treatment), retention time(s) of gas chromatography, and peak intensities, respectively.

**Figure 2:**
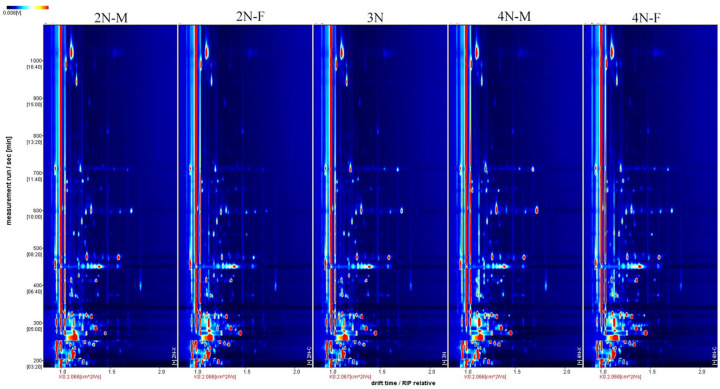
Two-dimensional top view of five groups of volatile matter in oysters. The *x*-axis and *y*-axis indicate the migration time (normalized treatment), and retention time(s), respectively.

The three-dimensional spectra of volatile components within 2N-M, 2N-F, 3N, 4N-M, and 4N-F oyster groups were evaluated using GC-IMS (Figure 1). The *x*-axis, *y*-axis, and *z*-axis in Figure 1 indicate the migration time (normalized treatment), retention time(s) of gas chromatography, and peak intensities, respectively. The distributions of peak signals were highly similar across groups, indicating overall similarity in VOCs across male and female oysters with different ploidy levels. However, some differences in peak intensities were observed among samples, indicating differences in volatile component contents among oysters. To more directly assess differences in the types and concentrations of volatile substances in each sample, the three-dimensional GC-IMS spectra were projected onto a two-dimensional plane to obtain an overhead GC-IMS plane (Figure 2). The red vertical line at an *x*-axis value of 1.0 (Figure 2) represents the normalized active ion peak (RIP). Each point on both sides of the ion peak represents one VOC and the depth of the color indicates concentration levels, with higher concentrations in red and lower concentrations in white.

**Figure 3:**
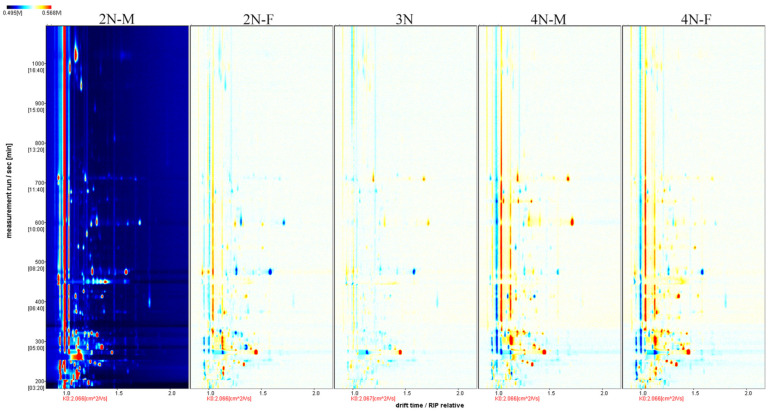
Comparison and differences in the spectra of volatile components in the five groups of oysters. Using the 2N-M sample as a reference, the comparison shows how all the volatile substances in the sample differ from one sample to another, with red representing a higher concentration of the substance in the sample than the reference sample and blue representing a lower concentration.

To more accurately compare differences among the five oyster groups, the difference comparison model was used for the differential analysis of oyster samples. The composition profiles of the 2N-M oyster group were used as a reference in Figure 3, and the composition profiles of the other four groups were inferred from the 2N-M composition profiles. If the volatile components in the other samples were consistent with those in the 2N-M oysters, the two were offset and the background was white. In contrast, if the composition was higher than in the reference oyster, the value was colored red, or otherwise was shown in blue. Compared with diploid oysters, greater red points were apparent for triploid and tetraploid oysters during the retention period of 200–400 s and 600–800 s, indicating an enhanced signal of VOCs among oysters with different ploidy levels. Thus, significant differences in VOCs were present among oysters with different ploidy levels. Compared with the 2N-M oysters, more blue points were apparent for the 2N-F oysters during the retention period of 200–600 s. A similar observation was made for the tetraploid oyster samples, indicating that some volatile compounds disappeared, or the signal intensity decreased for oysters of different sexes. Thus, differences in VOCs between oysters of different sexes, but with the same ploidy level, were observed.

### 2.2. Identification of VOCs in Male and Female Oysters with Different Ploidy Levels

**Figure 4:**
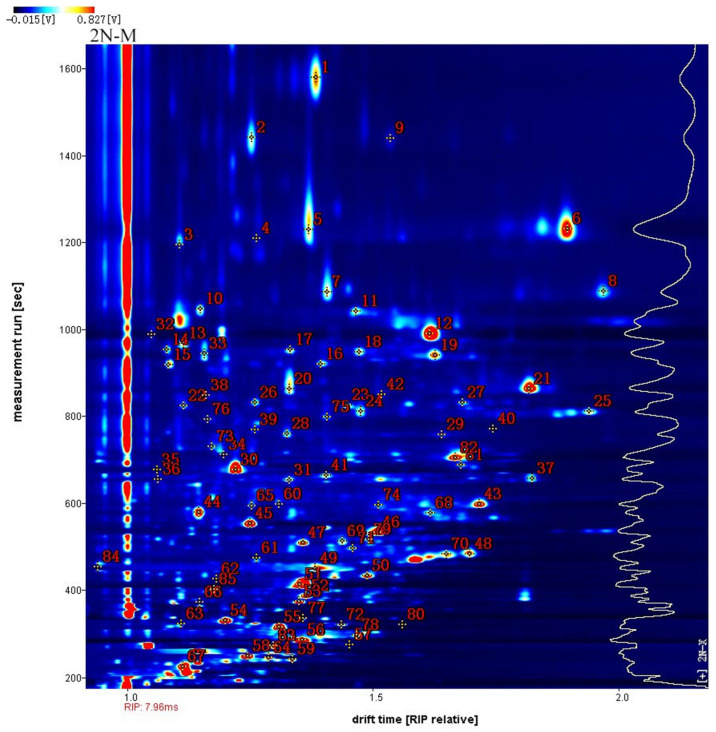
Qualitative analysis of different oyster samples by GC-IMC with 2N-M was taken as an example. The figures in the figure correspond to those in Table 1.

A qualitative analysis of the volatile components of oysters was conducted in this study (Figure 4). Taking 2N-M as an example, the *x*-axis represents the normalized ion migration time and the *y*-axis represents the retention time. Each point in Figure 4 represents a compound that was individually identified (Table 1). Eighty-five peaks were detected among the five groups, comprising fifty-four volatile compounds that accounted for 92.25% of the total peak area. These volatiles consisted of twenty alcohols, thirteen ketones, eight alcohols, five esters, two acids, three furans, two pyridines, and one ether. Due to the different concentrations of the compounds, several signals were generated when the concentrations of some compounds were too high. Thus, multiple spots were generated to visualize multiple peaks (indicating the production of monomers and dimers), when passing through the drift region. Qualitative results are shown in Table 1. Some of these compounds exhibited multiple peaks, including phenylacetaldehyde, propanoic acid, (E)-non-3-en-2-one, (E)-2-nonenal, benzaldehyde, furfural, 3-(methylsulfonyl)propanal, (E)-2-octenal, (E,E)-2,4-hexadienal, 1-nonanal, 1-hexanol, 2-butanone, 3-hydroxy, 3-octanone, (Z)-4-heptenal, and 1-pentanol, indicating the presence of both monomers and dimers of these compounds.

**Figure 5:**
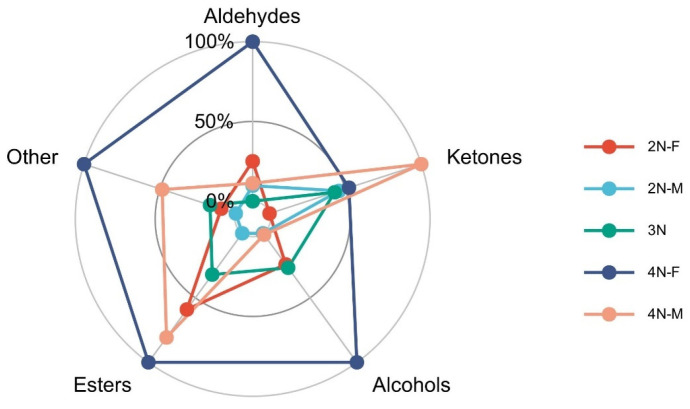
Comparison of VOCs in oysters from different samples.

Ketones (38.4%), aldehydes (14.26%), alcohols (18.5%), and esters (12.8%) were the primary components of the volatile aromatic compounds detected in the oyster samples. Differences in VOCs from different samples are shown in Figure 5. Compared with diploid and triploid oysters, tetraploid oysters exhibited significantly increased levels of volatile components (*p* < 0.05), indicating that oysters with different ploidy levels greatly differed in flavor profiles. Thus, changes in ploidy also resulted in altered compositions of volatile components. In addition, the ketone levels in 2N-M oysters were significantly higher than in 2N-F oysters, while levels of aldehydes, alcohols, and esters were significantly higher in 2N-F oysters than in 2N-M oysters. The same observation was also made for tetraploid oysters, indicating differences in volatile flavor compounds between male and female oysters.

Ketones were the primary volatile flavor compounds that contributed to the oyster profiles. Ketones are produced by the oxidation or degradation of polyunsaturated fatty acids, the degradation of amino acids, or microbial oxidation, but they may also be generated by the oxidation of alcohols and decomposition of esters [23]. Ketones are also more stable than other volatile substances and are less prone to oxidation, resulting in longer-lasting floral fragrances. Among the ketones, 3-pentanone, 2-propanone, and 1-penten-3-one significantly contributed to the volatile flavor profiles of oysters, including through sweet floral and fruit-scented odors.

A total of 20 aldehydes were detected, and these were the most diverse compounds identified in this study. Aldehydes are typically produced by the oxidative degradation of polyunsaturated fatty acids [24]. The primary aldehydes identified here were (E)-2-pentenal, butanal, phenylacetaldehyde, (E)-2-nonenal, and (E)-2-butenal. Aldehydes generally have a low odor threshold and play an important role in oyster biological processes, while greatly influencing the flavors of oysters. Among the aldehydes, olefine aldehyde and enal contents were very high. (E)-2-pentenal produces a grass-like smell [19], while (E)-2-octena can produce an almond, cucumber-like taste [25] and phenylacetaldehyde can produce a sweet honey-like taste [26]. Benzaldehyde was also detected; when volatilized, it produces a bitter almond taste that may be related to amino acid degradation [27].

Alcohol compound thresholds are relatively high, and these compounds come from the oxidation and decomposition of oils [28] that do not appreciably contribute to the flavors of shellfish substances. However, the threshold value for unsaturated aldehydes is relatively low, and these compounds greatly contribute to oyster flavors. Alcohols often produce aromatic and plant-based aromas [2]. Among them, 1-octen-3-ol, 1-butanol, 1-penten-3-ol, and 1-propanol contribute most to oyster flavors. 1-octen-3-ol can produce unique earthy and mushroom tastes, primarily from the oxidation of unsaturated fatty acids [29].

Esters are obtained by the esterification of alcohols and carboxylic acids [30], exhibiting unique fruity and aromatic odors. Detected ester compounds included butyl pentanoate, ethyl propanoate, ethyl acetate, ethyl (E)-2-butenoate, and ethyl-butyrate.

Among the acids, propionic and acetic acids were detected at a low content, although their threshold values are large that they minimally affect oyster flavors. Furans are primarily generated by sugar decomposition and Maillard reactions, exhibit a very low aroma threshold, and imbue caramel- or meat-like odors, among others. Maillard reactions can occur between amino acids and reducing sugars, resulting in the production of numerous important flavor compounds, including heterocyclic compounds such as furans and pyrazine [31]. Heterocyclic compounds that were identified included 2-ethyl furan, 2-butylfuran, 2-pentyl furan, 2-ethyl-3-methyl pyrazine, and 2,3,5-trimethyl pyrazine, which can all produce attractive aromatic flavors. Overall, the odor profiles from the above compounds contribute to the volatile odors of di-, tri-, and tetraploid oysters being recognized as grassy, fatty, and sweet fruity aromas, respectively.

### 2.3. Fingerprint Analysis of VOCs in Male and Female Oysters with Different Ploidy Levels

**Figure 6:**
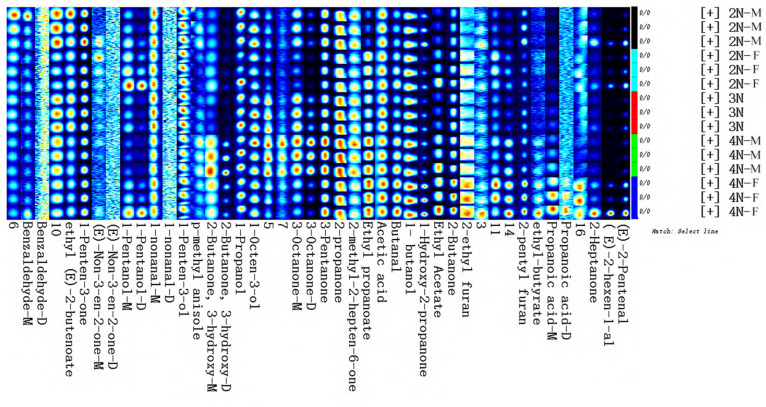
Oyster sample fingerprints after incubation at 40 °C. Each row represents all of the signal peaks that were chosen in a sample and each column depicts the signal peak of the same volatile chemicals in various oyster samples.

To more directly evaluate the variation in volatile compounds and their relative concentrations in different oyster samples, a fingerprint map of VOCs was visualized to quantitatively compare differences in VOCs among groups. The fingerprint profiles of oyster samples were generated based on peak volumes (Figure 6; rows represent volatile compositions and columns represent signal peaks of VOCs for the samples, while peak color correlates to VOC concentration, with color brightness correlated to higher concentrations). Compounds are denoted by numbers; nameless ones denote undefined substances, and the suffixes -M and -D correspond to monomers and dimers, respectively. The comparison of fingerprints allowed the visualization of dynamic changes in the VOCs. Although significant differences in the types of volatile compounds were not apparent, a significant difference in volatile compound contents was apparent among the samples, and volatile compound contents in tetraploid oysters were significantly higher. Benzaldehyde, ethyl(E)-2-butenoate, and 1-penten-3-one were most abundant in diploid male samples, while (E)-non-3-en-2-one and 1-pentanol were most abundant in diploid female samples, and 1-nonanal and 1-penten-3-ol were most abundant in triploid samples. In addition, 2-butanone, 3-hydroxy, 3-octanone, 3-pentanone, 2-propanone, and 2-methyl-2-heptene-6-one were most abundant in tetraploid male samples, while (E)-2-pentenal, (E)-2-hexen-1-al, propanoic acid, 2-heptanone, 2-pentyl furan, and 2-ethyl furan were most abundant in tetraploid female samples.

### 2.4. Principal Component Analysis (PCA) of Volatile Compounds Profiles

**Figure 7:**
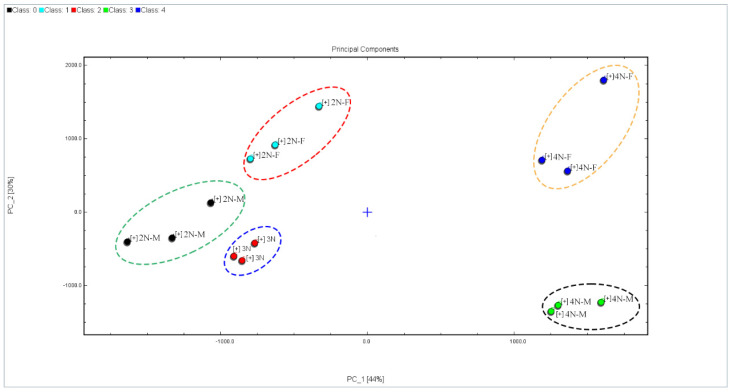
PCA of VFC profiles among different oysters.

To better visualize the sample data and remove noise, the dimensionality of the original data was reduced, followed by the classification of samples using PCA (Figure 7). PCA is a multivariate statistical method used to assess correlations between multiple variables and can be used to comprehensively evaluate multidimensional problems. Generally, when the cumulative contribution rates of PC1 and PC2 reach 60%, the PCA model can be used to adequately separate different samples. PCA was used to distinguish oysters with different ploidy profiles in this study, with contributions of PC1 and PC2 of 44% and 30%, respectively (total cumulative contribution of 74%), indicating distinct characteristic differences in flavor compounds between male and female oyster samples with different ploidy profiles. The samples were clearly separated in the ordination and the samples with high correlations were distributed in the same region (Figure 7). The tetraploids were clustered separately from the other samples, and exhibited a positive PC1 score, indicating that ploidy differences were associated with significant differences in VOC profiles. The VOC compositions of the diploids were relatively similar to those of the triploids, indicating the presence of more similar VFCs within diploid and triploid oysters compared to tetraploid oysters, as reflected also in more similar sensory characteristics. In addition, the 4N-M and 4N-F samples were clearly distinguished through PC2. Similar differences were observed for the diploid samples, indicating that gender differences can affect VFC profiles in oysters. Thus, GC-IMS techniques can effectively produce distinguishable VOC profiles of tetraploid and diploid oysters, triploid oyster samples, and different sexes of diploid/tetraploid oyster samples.

### 2.5. Comparison of Volatile Compounds between Different Sexes of Oysters

Differences in concentration of volatile compounds between diploid and tetraploid oysters of different sexes were also analyzed, based on compound peak volumes (Table 2). No significant differences in total volatile compounds were observed between diploid and tetraploid female and male oysters. Considering the edible components of diploid oysters, the contents of several volatile compounds were more abundant in diploid female oysters than in diploid male oysters (*p* < 0.05), including propanoic acid, (E,E)-2,4-hexadienal, 2-butanone,3-hydroxy, acetic acid, 2-butanone,3-hydroxy, 1-butanol, ethyl propanoate, butanal, 2-propanone, and 2-ethyl furan. Several volatile compounds were also significantly lower in females than in males (*p* < 0.05), including p-methyl anisole, (E)-2-octenal, 1-hydroxy-2-propanone, 3-octanone, 2-pentyl furan, 3-pentanone, 3-octanone, 2-nonanone, and (E)-2-heptenal. Several volatile compounds were identified that were significantly enriched in tetraploid female oysters compared to male oysters (*p* < 0.05), including propanoic acid, 1-hydroxy-2-propanone, 2-pentyl furan, 1-butanol, ethyl propanoate, 2-butanone, butanal, and 2-ethyl furan. In addition, several VOCs were significantly more abundant in male oysters than in female oysters, including p-methyl anisole, 2-butanone,3-hydroxy, 3-octanone, 3-octanone, ethyl (E)-2-butenoate, 2-propanone, 2-methyl-2-hepten-6-one, (E)-2-heptenal, and 2-butylfuran (*p* < 0.05). Overall, the volatile compounds in female oysters were significantly different from those in male oysters. The reason for the difference in volatile compounds in male and female oysters may be that the nutrients required by egg and sperm are different, which leads to the difference in volatile flavor compounds in the gonads of male and female oysters. Because the gonads’ primary role is to provide nutrients for gametogenesis and development, female oyster gonads are rich in lipids, while male oyster gonads are rich in protein. On the other hand, it could be related to the unique meiotic modes of oysters. Spermatozoa are involved in more cell membrane synthesis and require more energy, resulting in differences in biochemical composition and thus the flavor substances exhibiting differently.

### 2.6. Comparison of Volatile Compounds between Different Ploidy Oysters

Volatile compounds that resulted in the volatile odor differences between diploid and triploid oysters included eight ketones, four aldehydes, four esters, three alcohols, two acids, and one furan (Table 3). Ketones and aldehydes were the main factors that affected the volatile odor differences among diploid, triploid, and tetraploid oysters. Ketones were the most abundant, while aldehydes exhibited a low threshold and produced a significant effect on the aroma and taste sensory characteristics of oysters. The total content of the total volatile organic compounds (TVOC) in tetraploid oysters was significantly higher than in the diploid and triploid oysters (*p* < 0.05). Specifically, propanoic acid, 2-Butanone,3-hydroxy, Acetic acid, 2-Butanone,3-hydroxy, (Z)-4-heptenal, 1-butanol in tetraploid oysters, (E)-2-butenal, 3-Pentanone, Ethyl propanoate, 2-Butanone, Ethyl Acetate, Butanal, 2-propanone, ethyl-butyrate, and 2-ethyl furan content was significantly higher than that of diploidy and triploid (*p* < 0.05). Based on an analysis of detected volatile compounds, the diploid, triploid, and tetraploid oysters exhibited grassy, fatty, fruity, and sweet floral aromas on the whole, while these characteristic aromas were more prominent in tetraploid oysters than in diploid and triploid oysters. The reason for the difference in volatile flavor compounds may be that the cell size and shape caused by the different ploidy of *Crassostrea gigas* have physiological differences, and that change is probably related to the transcriptomes of different ploidy genome sizes, which affect the energy metabolism process of the oyster growth. The occurrence of gametes in oysters is inseparable from the component reserves of other tissues; triploids are sterile, and their energy is more used for growth, which leads to growth differences among triploids and tetraploid and diploids. *C. gigas* gonadal development is inseparable from glycogen, which is the main molecular contributor to the flavor quality of bivalves, which leads to differences among triploid-oyster and diploid- and tetraploid-oyster VOCs.

## 3. Materials and Methods

### 3.1. Materials

Fresh diploid, triploid and tetraploid *C. gigas* samples were collected in August of 2022 in Yantai city, Kong tong Island, Shandong Province. Diploid is a high-glycogen-content new strain Luyi No.1 (Certificate Number: GS-01-006-2020), which has been approved by the Ministry of Agriculture and Rural Affairs in China. Based on diploid Luyi No.1, we induced the tetraploid oyster, and triploid oysters were produced from diploid ♀ with tetraploid ♂ of Luyi No.1. We carefully opened the shell with a special knife, trying to keep it intact so as not to destroy the gonads. Firstly, we verified the ploidy status of each oyster using flow cytometry (Beckman Coulter Life Sciences, Shanghai, China). Single gill filaments of oysters were collected and immediately rinsed with PBS buffer to prevent contamination by the gonadal tissue from affecting the experimental results. The rinsed gill filaments were placed in ep tubes, followed by the addition of 300 μL of PBS buffer. They were cut up sufficiently, using scissors, to prepare a single cell suspension. A total of 10 μL of 0.05 mg/mL of DAPI (4′,6-Diamidino-2-phenylindole dihydrochloride) dye was added for staining, shaken well, and stained for 20 min, protected from light. Finally, the fixing solution was filtered through a 300 mesh filter screen, and the oyster ploidy was detected on the machine after completion. Afterward, the sex of the diploid and tetraploid oysters was detected, and a toothpick was used to scrape some of the material from the gonads, which was placed on a slide dripped with seawater and stirred gently. If it was grainy, it was a female oyster. If it was foggy or lumpy, it was a male oyster. The slide was placed under a microscope to confirm the gender again. After confirmation, they were divided into five groups: diploid male (2N-M), diploid female (2N-F), triploid (3N), tetraploid male (4N-M) and tetraploid female (4N-F). About 250g of soft tissue (the adductor muscle was excluded), with five oysters taken in each group. The homogenizer was used to homogenate the products, and they were put into a ziplocked bag and placed in a −80 °C refrigerator for freezing storage.

### 3.2. Experimental Methods

#### 3.2.1. GC-IMS Analysis

The volatile components of each sample were identified using the Flavor Spec^®^ flavor analyzer (G.A.S, IMSPEX Diagnostics Ltd., Rhondda Cynon Taff, UK). Prior to the experiment, the samples were thawed at 4 °C. Then, 2 g of samples from homogenates of the five oysters were weighed and placed into a bottle with 20 mL headspace, capped, and sealed. After incubating at 40 °C for 15 min, 700 μL samples were injected into the instrument with carrier gas through automatic headspace sampling. The incubation speed was 500 rpm and the injection needle temperature was 105 °C. After the initial separation using the gas chromatography column and then in the ion migration tube, the molecules to be measured migrated to the Faraday disc for detection under the action of the electric field; this was reversed by the drift gas after ionization in the ionization region, and secondary separation was achieved. Three parallel samples were included for each group.

#### 3.2.2. GC-IMS Analysis

An MXT-WAX metal chromatographic column was used for GC-IMS, which exhibited an inner diameter of 0.53 mm and a film thickness of 1 μm. The column temperature was set to 60 °C, the carrier gas/drift gas was dinitrogen (99.999% purity), and the temperature of the ion mobility spectrum was 45 °C. Carrier gas flow rates were set at 0–2 min, 2 mL/min; 2–10 min, 10 mL/min; 10–30 min, 100 mL/min, and a drift gas flow of 150 mL/min. The analysis time was 30 min.

#### 3.2.3. Data Analysis

The VOCal software (Version 0.4.03, GAS Deutschland, Dortmund, Germany) program was used to analyze the spectra and characterize the VOCs in the samples. The NIST (National Institute of Standards and Technology) and IMS (Information Management System) databases integrated into the software were used to identify the compounds. The Reporter plug-in was used to directly and easily compare the two-dimensional top views and three-dimensional spectra between the samples, enabling a comparison of the VOC profiles among samples. The Gallery Plot plug-in was used to visualize the fingerprint spectra of the volatile substances, followed by a qualitative and quantitative comparison of differences in the VOCs among the different samples. The Dynamic PCA (principal component analysis) plug-in was used for classification analysis to observe variation in the VOC compositions among the samples. The SPSS 20.0 software program (SPSS Inc., Chicago, IL, USA) was used to assess the significance of different ploidy and sex groups, based on the pairwise comparison method (Duncan) and by using a *p* < 0.05 significance threshold. The experimental results are presented as means ± standard deviation (mean ± SD).

## 4. Conclusions

In this study, volatile compound profiles were investigated among five groups of oysters, based on characteristic peak volumes determined with GC-IMS analysis. These analyses provided high resolution for understanding the differences in flavor compounds between oysters with different genders and ploidy levels. The total contents of volatile flavoring substances in the tetraploid oysters were significantly higher than in diploid and triploid oysters. Several compounds were significantly higher (*p* < 0.05), in tetraploid oysters than in diploid and triploid oysters, including propanoic acid, 2-butanone,3-hydroxy, acetic acid, 2-butanone,3-hydroxy, (Z)-4-heptenal, 1-butanol, (E)-2-butenal, 3-pentanone, ethyl propanoate, 2-butanone, ethyl acetate, butanal, 2-propanone, ethyl-butyrate, and 2-ethyl furan. Thus, the volatile aromatic characteristics of the tetraploid oysters were significantly different from those of the diploid and triploid oysters. Further, the contents of ethyl (E)-2-butenoate and 1-penten-3-ol were significantly higher in the edible parts of triploid oysters than in diploid and tetraploid oysters (*p* < 0.05). This could be related to triploid oysters using less energy for gonadal development. The concentrations of several volatile compounds in the female oysters were significantly higher than in the male oysters, including propanoic acid, ethyl propanoate, 1-butanol, butanal, and 2-ethyl furan. In contrast, p-methyl anisole, 3-octanone, 3-octanone, and (E)-2-heptenal concentrations were more abundant in male than female oysters. Differences between male and female oysters could be related to the oocytes of female oysters stagnating in the M1 phase of meiosis before egg laying, and may also have to do with the different nutrients that eggs and sperm need, leading to different concentrations in male and female gonads. Meanwhile, the differences among diploid, triploid and tetraploid oysters may be related to physiological differences in cell size and shape, related to the energy metabolism processes that affect oyster growth. In conclusion, this study analyzed differences in volatile flavor compounds between oysters of different ploidy and gender for the first time, providing new insights into our understanding of oyster flavor characteristics.

## Figures and Tables

**Table 1:** Qualitative GC-IMS results of male and female oysters with different ploidy oysters.

No.1	Compound	CAS#	Formula	MW ^a^	RI ^b^	Rt ^c^	Dt ^d^	Comment ^e^
1	(E, Z)-2,6-nonadienal	C557482	C_9_H_14_O	138.2	1737.5	1580.877	1.3853	
2	phenylacetaldehyde	C122781	C_8_H_8_O	120.2	1691.5	1441.904	1.25563	Monomer
3	Propanoic acid	C79094	C_3_H_6_O_2_	74.1	1597.9	1195.785	1.10796	Monomer
4	Propanoic acid	C79094	C_3_H_6_O_2_	74.1	1603.6	1209.576	1.26464	Dimer
5	(E)-Non-3-en-2-one	C18402830	C_9_H_16_O	140.2	1611.9	1229.733	1.37089	Monomer
6	(E)-Non-3-en-2-one	C18402830	C_9_H_16_O	140.2	1612.7	1231.854	1.89675	Dimer
7	(E) -2-nonenal	C18829566	C_9_H_16_O	140.2	1549.9	1086.517	1.40871	Monomer
8	(E) -2-nonenal	C18829566	C_9_H_16_O	140.2	1550.3	1087.265	1.96933	Dimer
9	phenylacetaldehyde	C122781	C_8_H_8_O	120.2	1691.2	1441.125	1.53643	Dimer
10	Benzaldehyde	C100527	C_7_H_6_O	106.1	1531.9	1048.106	1.1505	Monomer
11	Benzaldehyde	C100527	C_7_H_6_O	106.1	1529.3	1042.566	1.46691	Dimer
12	(E, E)-2,4-heptadienal	C4313035	C_7_H_10_O	110.2	1503.8	990.859	1.6172	
13	p-methyl anisole	C104938	C_8_H_10_O	122.2	1491.9	967.468	1.1157	
14	Furfural	C98011	C_5_H_4_O_2_	96.1	1484.8	953.925	1.08248	Monomer
15	3-(methylsulfanyl)propanal	C3268493	C_4_H_8_OS	104.2	1466.1	918.838	1.08564	Monomer
16	3-(methylsulfanyl)propanal	C3268493	C_4_H_8_OS	104.2	1467.4	921.301	1.39572	Dimer
17	Furfural	C98011	C_5_H_4_O_2_	96.1	1484.2	952.694	1.33244	Dimer
20	(E)-2-octenal	C2548870	C_8_H_14_O	126.2	1434.9	863.438	1.33086	Monomer
21	(E)-2-octenal	C2548870	C_8_H_14_O	126.2	1434.9	863.438	1.81812	Dimer
22	(E, E)-2,4-hexadienal	C142836	C_6_H_8_O	96.1	1411.6	824.042	1.1157	Monomer
23	(E, E)-2,4-hexadienal	C142836	C_6_H_8_O	96.1	1413.1	826.505	1.44793	Dimer
24	1-nonanal	C124196	C_9_H_18_O	142.2	1403.7	811.116	1.4764	Monomer
25	1-nonanal	C124196	C_9_H_18_O	142.2	1404	811.731	1.94152	Dimer
28	1 -hexanol	C111273	C_6_H_14_O	102.2	1370.7	759.409	1.32611	Monomer
29	1 -hexanol	C111273	C_6_H_14_O	102.2	1369.5	757.562	1.64093	Dimer
30	1-Hydroxy-2-propanone	C116096	C_3_H_6_O_2_	74.1	1313.6	677.539	1.2217	
31	2-Butanone, 3-hydroxy	C513860	C_4_H_8_O_2_	88.1	1294.8	652.276	1.3317	Dimer
32	Acetic acid	C64197	C_2_H_4_O_2_	60.1	1502.6	988.507	1.05136	
33	1-Octen-3-ol	C3391864	C_8_H_16_O	128.2	1479.7	944.205	1.1587	
36	2-Butanone, 3-hydroxy	C513860	C_4_H_8_O_2_	88.1	1296.6	654.891	1.06375	Monomer
37	1-octanal	C124130	C_8_H_16_O	128.2	1298.7	657.566	1.82562	
38	2-Ethyl-3-methyl pyrazine	C15707230	C_7_H_10_N_2_	122.2	1426.5	849.028	1.16209	
41	butyl pentanoate	C591684	C_9_H_18_O_2_	158.2	1304.3	665.014	1.40639	
43	3-Octanone	C106683	C_8_H_16_O	128.2	1265.7	598.497	1.71809	Dimer
44	(Z)-4-heptenal	C6728310	C_7_H_12_O	112.2	1254.7	579.319	1.14747	Monomer
45	2-pentyl furan	C3777693	C_9_H_14_O	138.2	1239	552.949	1.25109	
46	(E)-2-hexen-1-al	C6728263	C_6_H_10_O	98.1	1227.3	534.114	1.51085	
47	3-Methyl-2-butenal	C107868	C_5_H_8_O	84.1	1211.6	509.799	1.35897	
48	Heptaldehyde	C111717	C_7_H_14_O	114.2	1194.1	484.114	1.69822	
49	1-butanol	C71363	C_4_H_10_O	74.1	1169.8	450.824	1.38425	
50	(Z)-2-Methylpent-2-enal	C623369	C_6_H_10_O	98.1	1157.3	434.743	1.48969	
51	(E)-2-Pentenal	C1576870	C_5_H_8_O	84.1	1139.8	413.076	1.35223	
52	(Z)-2-pentenal	C1576869	C_5_H_8_O	84.1	1118	387.568	1.36614	
53	(E)-3-penten-2-one	C3102338	C_5_H_8_O	84.1	1105.9	374.154	1.35167	
54	(E)-2-butenal	C123739	C_4_H_6_O	70.1	1053.9	331.065	1.20029	
55	1-Penten-3-one	C1629589	C_5_H_8_O	84.1	1031.9	315.212	1.31048	
56	3-Pentanone	C96220	C_5_H_10_O	86.1	985.5	285.945	1.35723	
57	Ethyl propanoate	C105373	C_5_H_10_O_2_	102.1	961.9	274.969	1.45295	
58	2-Butanone	C78933	C_4_H_8_O	72.1	901	248.547	1.24704	
59	Ethyl Acetate	C141786	C_4_H_8_O_2_	88.1	884	241.637	1.33831	
60	3-Octanone	C106683	C_8_H_16_O	128.2	1265.2	597.553	1.30973	Monomer
62	ethyl (E)-2-butenoate	C623701	C_6_H_10_O_2_	114.1	1150.4	426.075	1.18305	
63	1-Propanol	C71238	C_3_H_8_O	60.1	1043.2	323.287	1.11199	
64	Butanal	C123728	C_4_H_8_O	72.1	896.2	246.591	1.28862	
65	1-Pentanol	C71410	C_5_H_12_O	88.1	1263.7	594.974	1.25483	Monomer
67	2-propanone	C67641	C_3_H_6_O	58.1	843.9	226.112	1.11453	
68	(Z)-4-heptenal	C6728310	C_7_H_12_O	112.2	1254	578.092	1.61885	Dimer
70	2-Heptanone	C110430	C_7_H_14_O	114.2	1193.4	483.056	1.65086	
73	2-methyl-2-hepten-6-one	C110930	C_8_H_14_O	126.2	1351.1	730.198	1.17369	
74	1-Pentanol	C71410	C_5_H_12_O	88.1	1264.4	596.137	1.51151	Dimer
75	2-Nonanone	C821556	C_9_H_18_O	142.2	1396.2	799.107	1.40828	
76	2,3,5- trimethylpyrazine	C14667551	C_7_H_10_N_2_	122.2	1393.1	794.156	1.16446	
79	1-Butanol, 3-methyl	C123513	C_5_H_12_O	88.1	1216.4	517.124	1.49232	
80	ethyl-butyrate	C105544	C_6_H_12_O_2_	116.2	1040.9	321.634	1.56149	
81	1-Octen-3-one	C4312996	C_8_H_14_O	126.2	1321.3	688.033	1.67936	
82	(E)-2-Heptenal	C18829555	C_7_H_12_O	112.2	1333.5	704.915	1.6689	
83	2-ethyl furan	C3208160	C_6_H_8_O	96.1	957.8	273.08	1.29858	
84	1-Penten-3-ol	C616251	C_5_H_10_O	86.1	1171.9	453.687	0.94218	
85	2-butylfuran	C4466244	C_8_H_12_O	124.2	1130.9	402.512	1.17811	

^a^ Represents the molecular mass. ^b^ Represents the retention index of volatile components calculated. ^c^ Represents the retention time in the capillary GC column (unit: s). ^d^ Represents the drift time in the tube (unit: msec). ^e^ Represents the volatile component, which was monomer or dimer.

**Table 2:** GC-IMS-based identification of differences between male and female oysters.

No.	Compound	The Peak Volume			
		2N-M	2N-F	4N-M	4N-F
3	Propanoic acid	104.56 ± 3.98 ^b^	122.28 ± 7.57 ^a^	-	-
		-	-	300.06 ± 49.44 ^b^	615.37 ± 103.41 ^a^
13	p-methyl anisole	175.79 ± 12.15 ^a^	94.12 ± 11.26 ^b^	-	-
		-	-	164.68 ± 15.94 ^a^	112.81 ± 22.24 ^b^
21	(E)-2-octenal	85.89 ± 7.44 ^a^	70.27 ± 10.64 ^b^	-	-
		-	-	88.06 ± 6.30	80.31 ± 14.13
23	(E,E)-2,4-hexadienal	43.16 ± 2.50 ^b^	56.04 ± 7.30 ^a^	-	-
		-	-	47.54 ± 10.81	45.02 ± 2.52
30	1-Hydroxy-2-propanone	477.89 ± 44.53 ^a^	324.98 ± 53.87 ^b^	-	-
		-	-	342.43 ± 28.23 ^b^	551.35 ± 21.73 ^a^
31	2-Butanone,3-hydroxy	72.97 ± 7.12 ^b^	93.46 ± 8.48 ^a^	-	-
		-	-	234.25 ± 55.86	155.55 ± 15.99
32	Acetic acid	1576.30 ± 5.72 ^b^	1652.99 ± 16.02 ^a^	-	-
		-	-	1760.70 ± 45.02	1825.28 ± 37.38
36	2-Butanone,3-hydroxy	95.25 ± 6.71 ^b^	129.73 ± 12.40 ^a^	-	-
		-	-	235.27 ± 24.64 ^a^	176.28 ± 9.61 ^b^
43	3-Octanone	465.51 ± 16.49 ^a^	165.57 ± 2.74 ^b^	-	-
		-	-	1676.19 ± 155.93 ^a^	450.18 ± 70.83 ^b^
45	2-pentyl furan	130.04 ± 8.65 ^a^	99.22 ± 13.44 ^b^	-	-
		-	-	114.70 ± 21.40 ^b^	163.59 ± 12.81 ^a^
49	1-butanol	1062.35 ± 26.96 ^b^	1165.43 ± 13.89 ^a^	-	-
		-	-	1227.71 ± 80.73 ^b^	1434.19 ± 13.50 ^a^
56	3-Pentanone	3463.02 ± 33.01 ^a^	2435.40 ± 75.58 ^b^	-	-
		-	-	4518.05 ± 266.45	3509.57 ± 578.61
57	Ethyl propanoate	815.47 ± 52.01 ^b^	2568.56 ± 54.98 ^a^	-	-
		-	-	3093.35 ± 40.73 ^b^	3523.66 ± 107.29 ^a^
58	2-Butanone	745.20 ± 34.15	794.30 ± 46.05	-	-
		-	-	893.98 ± 33.19 ^b^	1112.21 ± 10.28 ^a^
60	3-Octanone	850.65 ± 37.88 ^a^	417.32 ± 36.7 ^b^	-	-
		-	-	1300.20 ± 43.89 ^a^	652.47 ± 111.80 ^b^
62	ethyl (E)-2-butenoate	379.92 ± 68.51	308.87 ± 4.05	-	-
		-	-	284.01 ± 5.25 ^a^	264.81 ± 7.75 ^b^
64	Butanal	330.81 ± 15.83 ^b^	472.97 ± 56.93 ^a^	-	-
		-	-	558.10 ± 48.26 ^b^	662.81 ± 35.06 ^a^
67	2-propanone	2616.37 ± 52.58 ^a^	2328.45 ± 134.30 ^b^	-	-
		-	-	3054.40 ± 95.93 ^a^	2775.62 ± 66.91 ^b^
73	2-methyl-2-hepten-6-one	78.51 ± 6.50	67.43 ± 4.05	-	-
		-	-	82.51 ± 4.17 ^a^	66.90 ± 5.42 ^b^
75	2-Nonanone	59.67 ± 4.50 ^a^	44.86 ± 3.27 ^b^	-	-
		-	-	54.25 ± 7.59	53.33 ± 7.33
82	(E)-2-Heptenal	76.38 ± 6.70 ^a^	57.71 ± 6.00 ^b^	-	-
		-	-	332.24 ± 18.93 ^a^	116.55 ± 27.76 ^b^
83	2-ethyl furan	40.83 ± 7.08 ^b^	71.54 ± 2.00 ^a^	-	-
		-	-	81.72 ± 2.01 ^b^	95.48 ± 5.88 ^a^
85	2-butylfuran	15.21 ± 1.43	13.14 ± 1.62	-	-
		-	-	53.36 ± 6.71 ^a^	20.86 ± 9.20 ^b^
TVOC ^f^		27,027.36 ± 1521.508	26,771.64 ± 2015.42	-	-
		-	-	34,016.38 ± 953.05	34,890.89 ± 2916.28

^f^ Total volatile organic compounds. Different letters in the same row indicate statistical differences between samples at a *p* < 0.05 level.

**Table 3:** Identification of VOC differences between oysters with different ploidy levels, based on GC-IMS analysis.

No.	Compound	The Peak Volume				
		2N-M	2N-F	3N	4N-M	4N-F
3	Propanoic acid	104.56 ± 3.98 ^c^	-	190.68 ± 30.05 ^b^	300.06 ± 49.44 ^a^	-
		-	122.28 ± 7.57 ^b^	190.68 ± 30.05 ^b^	-	615.37 ± 103.41 ^a^
15	3-(methylsulfanyl)propanal	92.75 ± 0.69 ^b^	-	104.51 ± 3.62 ^a^	103.67 ± 5.93 ^a^	-
		-	92.71 ± 4.40 ^b^	104.51 ± 3.62 ^a^	-	102.83 ± 0.82 ^a^
30	1-Hydroxy-2-propanone	477.89 ± 44.53 ^a^	-	311.82 ± 42.53 ^b^	342.43 ± 28.23 ^b^	-
		-	324.98 ± 53.87 ^b^	311.82 ± 42.53 ^b^	-	551.35 ± 21.73 ^a^
31	2-Butanone,3-hydroxy-D	72.97 ± 7.12 ^b^	-	64.33 ± 10.64 ^b^	234.25 ± 55.86 ^a^	-
		-	93.46 ± 8.48 ^b^	64.33 ± 10.64 ^c^	-	155.55 ± 15.99 a
32	Acetic acid	1576.30 ± 5.72 ^c^	-	1641.73 ± 2.81 ^b^	1760.70 ± 45.02 ^a^	-
		-	1652.99 ± 16.02 ^b^	1641.73 ± 2.81 ^b^	-	1825.28 ± 37.38 ^a^
36	2-Butanone,3-hydroxy	95.25 ± 6.71 ^b^	-	90.66 ± 16.97 ^b^	235.27 ± 24.64 ^a^	-
		-	129.73 ± 12.40 ^b^	90.66 ± 16.97 ^c^	-	176.28 ± 9.61 ^a^
43	3-Octanone	465.51 ± 16.49 ^c^	-	700.41 ± 42.92 ^b^	1676.19 ± 155.93 ^a^	-
		-	165.57 ± 2.74 ^c^	700.41 ± 42.92 ^a^	-	450.18 ± 70.83 ^b^
44	(Z)-4-heptenal	111.71 ± 38.42 ^b^	-	100.00 ± 21.30 ^b^	202.99 ± 5.99 ^a^	-
		-	144.08 ± 37.23 ^b^	100.00 ± 21.30 ^b^	-	244.71 ± 48.97 ^a^
49	1-butanol	1062.35 ± 26.96 ^b^	-	1129.44 ± 34.33 ^ab^	1227.71 ± 80.73 ^a^	-
		-	1165.43 ± 13.89 ^b^	1129.44 ± 34.33 ^b^	-	1434.19 ± 13.50 ^a^
54	(E)-2-butenal	209.26 ± 22.19 ^b^	-	202.55 ± 22.17 ^b^	328.25 ± 25.85 ^a^	-
		-	278.55 ± 107.07 ^b^	202.55 ± 22.17 ^b^	-	521.75 ± 157.66 ^a^
56	3-Pentanone	3463.02 ± 33.01 ^b^	-	2957.40 ± 98.31 ^c^	4518.05 ± 266.45 ^a^	-
		-	2435.40 ± 75.58 ^b^	2957.40 ± 98.31 ^ab^	-	3509.57 ± 578.61 ^a^
57	Ethyl propanoate	815.47 ± 52.01 ^c^	-	1969.56 ± 54.98 ^b^	3093.35 ± 40.73 ^a^	-
		-	2568.56 ± 54.98 ^b^	1969.56 ± 54.98 ^c^	-	3523.66 ± 107.29 ^a^
58	2-Butanone	745.20 ± 34.15 ^b^	-	879.82 ± 49.96 ^a^	893.98 ± 33.19 ^a^	-
		-	794.30 ± 46.05 ^c^	879.82 ± 49.96 ^b^	-	1112.21 ± 10.28 ^a^
59	Ethyl Acetate	532.94 ± 31.52 ^b^	-	593.64 ± 57.28 ^b^	1378.32 ± 360.09 ^a^	-
		-	1057.36 ± 317.92 ^b^	593.64 ± 57.28 ^b^	-	1636.02 ± 316.90 ^a^
60	3-Octanone-M	850.65 ± 37.88 ^c^	-	1052.75 ± 32.04 ^b^	1300.20 ± 43.89 ^a^	-
		-	417.32 ± 36.7 ^c^	1052.75 ± 32.04 ^a^	-	652.47 ± 111.80 ^b^
62	ethyl (E)-2-butenoate	379.92 ± 68.51 ^a^	-	371.75 ± 4.54 ^a^	284.01 ± 5.25 ^b^	-
		-	308.87 ± 4.05 ^b^	371.75 ± 4.54 ^a^	-	264.81 ± 7.75 ^c^
64	Butanal	330.81 ± 15.83 ^b^	-	387.23 ± 19.72 ^b^	558.10 ± 48.26 ^a^	-
		-	472.97 ± 56.93 ^b^	387.23 ± 19.72 ^c^	-	662.81 ± 35.06 ^a^
67	2-propanone	2616.37 ± 52.58 ^c^	-	2812.23 ± 76.19 ^b^	3054.40 ± 95.93 ^a^	-
		-	2328.45 ± 134.30 ^b^	2812.23 ± 76.19 ^a^	-	2775.62 ± 66.91 ^a^
80	ethyl-butyrate	14.77 ± 2.37 ^ab^	-	13.38 ± 3.50 ^b^	19.84 ± 1.30 ^a^	-
		-	14.09 ± 2.05 ^b^	13.38 ± 3.50 ^b^	-	24.56 ± 6.10 ^a^
82	(E)-2-Heptenal	76.38 ± 6.70 ^c^	-	185.52 ± 12.88 ^b^	332.24 ± 18.93 ^a^	-
		-	57.71 ± 6.00 ^c^	185.52 ± 12.88 ^a^	-	116.55 ± 27.76 ^b^
83	2-ethyl furan	40.83 ± 7.08 ^c^	-	59.79 ± 2.36 ^b^	81.72 ± 2.01 ^a^	-
		-	71.54 ± 2.00 ^b^	59.79 ± 2.36 ^c^	-	95.48 ± 5.88 ^a^
84	1-Penten-3-ol	1733.78 ± 17.79 ^b^	-	1821.35 ± 9.59 ^a^	1604.56 ± 20.70 ^c^	-
		-	1744.23 ± 8.94 ^b^	1821.35 ± 9.59 ^a^	-	1649.95 ± 25.41 ^c^
TVOC		27,027.36 ± 1521.50 ^b^	-	28,222.26 ± 714.81 ^b^	34,016.38 ± 953.05 ^a^	-
		-	26,771.64 ± 2015.46 ^b^	28,222.26 ± 714.81 ^b^	-	34,890.89 ± 2916.28 ^a^

Different letters in the same row indicate statistical differences among samples at a *p* < 0.05 level.

## Data Availability

Not applicable.

## Sample Availability

Samples of the compounds are not available from the authors.
